# Supplementary material for: A Critical View of the Application of the APEX Software (Aqueous Photochemistry of Environmentally-Occurring Xenobiotics) to Predict Photoreaction Kinetics in Surface Freshwaters
Source: Molecules. 2019 Dec 18;25(1):9. doi: 10.3390/molecules25010009 (PMC7017383; doi:10.3390/molecules25010009)
Supplement: Supplementary file 1 [file molecules-25-00009-s001.zip › 3.2.4_gcc-4.4.0.zip!/3.2.4_gcc-4.4.0/3.2.4_gcc-4.4.0/doc/octave/html/interpreter/Variable_002dlength-Return-Lists.html]

Variable-length Return Lists - Untitled


Next: Returning From a Function,
Previous: Variable-length Argument Lists,
Up: Functions and Scripts


---

### 11.4 Variable-length Return Lists

It is possible to return a variable number of output arguments from a
function using a syntax that's similar to the one used with the
special `varargin` parameter name. To let a function return a
variable number of output arguments the special output parameter name
`varargout` is used. As with `varargin`, `varargout` is
a cell array that will contain the requested output arguments.

As an example the following function sets the first output argument to
1, the second to 2, and so on.

```
     function varargout = one_to_n ()
       for i = 1:nargout
         varargout{i} = i;
       endfor
     endfunction
```

When called this function returns values like this

```
     [a, b, c] = one_to_n ()
          => a =  1
          => b =  2
          => c =  3
```

If `varargin` (`varargout`) does not appear as the last
element of the input (output) parameter list, then it is not special,
and is handled the same as any other parameter name.

— Function File: [r1, r2, ..., rn] = **deal** (a)  
— Function File: [r1, r2, ..., rn] = **deal** (a1, a2, ..., an)  
> Copy the input parameters into the corresponding output parameters.
> If only one input parameter is supplied, its value is copied to each
> of the outputs.
>
> For example,
>
> ```
>           [a, b, c] = deal (x, y, z);
> ```
>
> is equivalent to
>
> ```
>           a = x;
>           b = y;
>           c = z;
> ```
>
> and
>
> ```
>           [a, b, c] = deal (x);
> ```
>
> is equivalent to
>
> ```
>           a = b = c = x;
> ```
